# Brain Na^+^, K^+^-ATPase Activity In Aging and Disease

**Published:** 2014-06

**Authors:** Georgina Rodríguez de Lores Arnaiz, María Graciela López Ordieres

**Affiliations:** 1 Instituto de Biología Celular y Neurociencias “Prof. E. De Robertis”, CONICET-UBA Facultad de Medicina, Universidad de Buenos Aires, Paraguay 2155, 1121-Buenos Aires, Argentina;; 2 Cátedra de Farmacología, Facultad de Farmacia y Bioquímica, Junín 956, Universidad de Buenos Aires, 1113-Buenos Aires, Argentina.

**Keywords:** Na^+^, K^+^-ATPase during aging, Na^+^, K^+^-ATPase and neurological diseases, Na^+^, K^+^-ATPase and pathological states, Na^+^, K^+^-ATPase, Alzheimer disease

## Abstract

Na^+^/K^+^ pump or sodium- and potassium-activated adenosine 5’-triphosphatase (Na^+^, K^+^-ATPase), its enzymatic version, is a crucial protein responsible for the electrochemical gradient across the cell membranes. It is an ion transporter, which in addition to exchange cations, is the ligand for cardenolides. This enzyme regulates the entry of K^+^ with the exit of Na^+^ from cells, being the responsible for Na^+^/K^+^ equilibrium maintenance through neuronal membranes. This transport system couples the hydrolysis of one molecule of ATP to exchange three sodium ions for two potassium ions, thus maintaining the normal gradient of these cations in animal cells. Oxidative metabolism is very active in brain, where large amounts of chemical energy as ATP molecules are consumed, mostly required for the maintenance of the ionic gradients that underlie resting and action potentials which are involved in nerve impulse propagation, neurotransmitter release and cation homeostasis. Protein phosphorylation is a key process in biological regulation. At nervous system level, protein phosphorylation is the major molecular mechanism through which the function of neural proteins is modulted in response to extracellular signals, including the response to neurotransmitter stimuli. It is the major mechanism of neural plasticity, including memory processing. The phosphorylation of Na^+^, K^+^-ATPase catalytic subunit inhibits enzyme activity whereas the inhibition of protein kinase C restores the enzyme activity. The dephosphorylation of neuronal Na^+^, K^+^-ATPase is mediated by calcineurin, a serine / threonine phosphatase. The latter enzyme is involved in a wide range of cellular responses to Ca^2+^ mobilizing signals, in the regulation of neuronal excitability by controlling the activity of ion channels, in the release of neurotransmitters and hormones, as well as in synaptic plasticity and gene transcription. In the present article evidence showing Na^+^, K^+^-ATPase involvement in signaling pathways, enzyme changes in diverse neurological diseases as well as during aging, have been summarized. Issues refer mainly to Na^+^, K^+^-ATPase studies in ischemia, brain injury, depression and mood disorders, mania, stress, Alzheimer´s disease, learning and memory, and neuronal hyperexcitability and epilepsy.

## INTRODUCTION

Na^+^/K^+^ pump or sodium- and potassium-activated adenosine 5’-triphosphatase (Na^+^, K^+^-ATPase), its enzymatic version, is a crucial protein responsible for the electrochemical gradient across the cell membranes. It is an ion transporter, which in addition to exchange cations, is the ligand for cardenolides. This enzyme discovered by Skou (1) is essential to establish and maintain high K^+^ and low Na^+^ concentration in the cytoplasm. It regulates the entry of K^+^ with the exit of Na^+^ from cells. Therefore, it is responsible for Na^+^ / K^+^ equilibrium maintenance through neuronal membranes. The impairment of such equilibria leads to nerve ending depolarization with Ca^2+^ entry to the cell. This is followed by neurotransmitter release and neuronal swelling, which is obviously detrimental to cell function. This transport system couples the hydrolysis of one molecule of ATP to exchange three sodium ions for two potassium ions, thus maintaining the normal gradient of these cations in animal cells (2, 3).

Na^+^, K^+^-ATPase is a membrane-bound enzyme which is critical in neurons for the regulation of membrane potential, cell volume and transmembrane fluxes of Ca^2+^ and excitatory neurotransmitters. It is also crucial in the normal cell cycle and differentiation of the nervous system. In specialized cells, the maintenance of Na^+^ and K^+^ gradients between the intracellular and extracellular compartments is a prerequisite for basic cellular homeostasis and for diverse functions (3).

The activity of neuronal Na^+^, K^+^-ATPase concentrates in the surrounding nerve ending membranes. In these membranes specific receptors for classical neurotransmitter and neuropeptides are also inserted (4-6). All these macromolecules are most likely localized contiguously and therefore may well interact at central nervous system (CNS). The possibility of their regulation by released active substances at synapses seems tenable.

It is known that oxidative metabolism is very active in brain, where large amounts of chemical energy as ATP molecules are consumed, mostly required for the maintenance of the ionic gradients that underlie resting and action potentials which are involved in nerve impulse propagation, neurotransmitter release and cation homeostasis (3).

Na^+^, K^+^-ATPase is the pharmacological receptor for cardiotonic steroids such as ouabain and digoxin, which behave as enzyme inhibitors (7). Cardiotonic steroids are synthesized in the brain (8) and are present in the hypothalamus of Milan hypertensive rats (7). A 3D-structural model of the Na^+^, K^+^-ATPase digitalis binding site has been constructed (9).

To maintain neuronal cytoplasmic Ca^2+^ concentration one-ten thousand times lower that in the extracellular millieu, two mechanisms are involved: a calcium pump and a Na^+^ / Ca^2+^ exchanger. The latter depends on functional Na^+^, K^+^-ATPase, in a process inhibited by omitting Na^+^ and including ouabain. Therefore, failure of the Na^+^, K^+^-pump produces depletion of intracellular K^+^, accumulation of intracellular free Ca^2+^ by activation of voltage-gated Ca^2+^ channels and reversion of the Na^+^ / Ca^2+^ exchanger (10).

## Na^+^, K^+^-ATPase REGULATION BY PHOSPHORYLATION/DEPHOSPHORYLATION

It is known that protein phosphorylation is a key process in biological regulation. It involves a protein kinase, a protein phosphatase and a substrate protein. The kinases catalyze the transfer of the terminal γ phosphate of ATP to the hydroxyl moiety in the correponding amino acid residue, in a reaction which requires Mg^2+^. In turn, the protein phosphatases catalyze the cleavage of this phosphoester bond through hydrolysis. Phosphorylation of diverse protein types is involved in regulating or in carrying out nervous system processes. At nervous system level, protein phosphorylation is the major molecular mechanism through which the function of neural proteins is regulated in response to extracellular signals, including the response to neurotransmitter stimuli. Indeed, it is the major mechanism of neural plasticity, including memory processing. Regarding the subject of the present article, it should be recalled that Na^+^, K^+^-ATPase is a phosphorylation substrate (11).

The phosphorylation of Na^+^, K^+^-ATPase catalytic subunit inhibits enzyme activity (12). Accordingly, inhibition of protein kinase C (PKC) restores the enzyme activity (13, 14). The dephosphorylation of neuronal Na^+^, K^+^-ATPase is mediated by calcineurin, a serine / threonine phosphatase. The latter enzyme is involved in a wide range of cellular responses to Ca^2+^ mobilizing signals, in the regulation of neuronal excitability by controlling the activity of ion channels, in the release of neurotransmitters and hormones, as well as in synaptic plasticity and gene transcription (15, 16). Besides, changes in dopamine- and cAMP-regulated phosphoprotein of 32 kDa (DARPP-32) activity may well lead to altered protein phosphatase 1 activity and hence to modified Na^+^, K^+^-ATPase dephosphorylation (17).

## BRAIN Na^+^, K^+^-ATPase STRUCTURE

**Figure 1:**
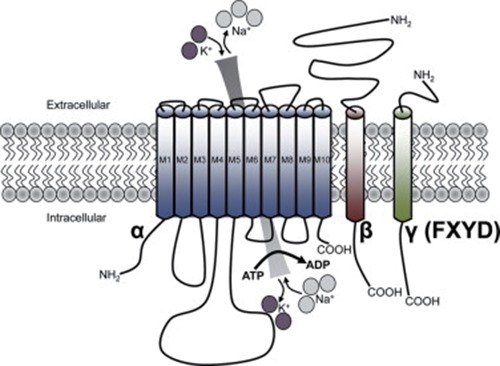
Schematic representation of Na^+^, K^+^-ATPase structure. The enzyme is a heterodimeric membrane spanning protein which is composed by α and β subunits, and, in some cases the γ (FXYD) subunit. The α subunit contains ten transmembrane domains whereas the β and γ subunits contain a single transmembrane domain. From reference 131, with permission.

Na^+^, K^+^-ATPase is an olygomeric enzyme consisting of α and β subunits, both required for enzyme function (Fig. 1). Alpha subunit is the catalytic one which exists in different isoforms: α1, α2, α3, or α4, the latter identified only in testis (18). The binding sites for ATP and the inhibitor ouabain as well as ion occlusion occur in α subunit (19). In the brain there are present isoforms α1, α2 and α3 which have cell-type and development-specific expression patterns. Subunits α1, α2, and α3 bind inhibitor ouabain with low, intermediate and high affinity, respectively. In neurons are present the last two isoforms whereas in glial cells are localized α1 and α2 isoforms (20-23).

The α2 isoform is widely expressed in neurons in late gestation but it is primarily expressed in astrocytes in adult brain. Most interesting, mice lacking the α2 isoform do not survive after birth (24).

Na^+^, K^+^-ATPase activity is regulated by some neurotransmitters (25-27). Such regulation is dependent on ontogeny (28). It is worthwhile to recall that all three α-subunit isoforms are present in neurons from the neostriatum and isoform specificity for neurotransmitter-dependent regulation of Na^+^, K^+^-ATPase activity has been suggested (29).

The β subunit regulates both the activity and the conformational stability of α subunit (30, 31) and seems to be involved in the modulation of enzyme affinity for K^+^ and Na^+^ (1, 33). It is important for ATP hydrolysis, ion transport, and binding of inhibitors such as ouabain. The β subunit must interact with α subunit in order to accomplish ion transport (2).

In association with the αβ dimmer there is a third subunit (γ) which belongs to the FXYD family proteins. This subunit modulates transport function of the enzyme (34), seems not essential for functional Na^+^, K^+^-ATPase but most likely plays a regulatory role in a tissue-specific manner (2, 35). The mammalian FXYD proteins from FXYD1 to FXYD7 exhibit tissue-specific distribution (36). They are considered to be regulators of ion channels or channels themselves. The function of these proteins is to modulate Na^+^, K^+^-ATPase catalytic properties by molecular interactions with specific enzyme domains (37, 38) (Fig. 1).

It is known that plasma membrane expression of the Na^+^, K^+^-ATPase requires the assembly of its α- and β- subunits. There is an interaction between the Na^+^, K^+^-ATPase α-subunit and the coat protein, β-COP, a component of the COP-1 complex. In the absence of the β- subunit the Na^+^, K^+^-ATPase α-subunit interacts with β-COP, is retained in the endoplasmic reticulum and targeted for degradation. In the presence of the β- subunit the α-subunit traffics directly to the plasma membrane (39).

## Na^+^, K^+^-ATPase AND SIGNALING PATHWAYS

**Figure 2:**
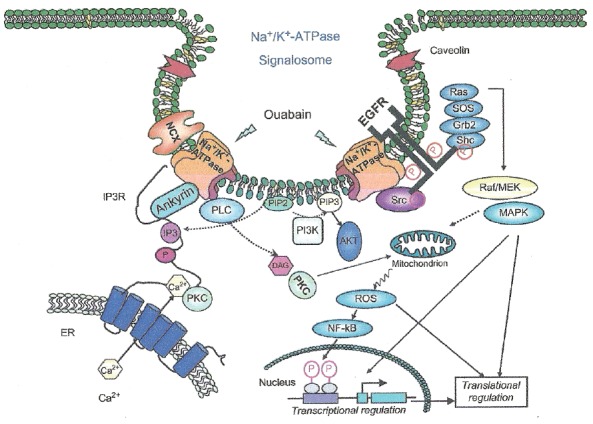
Na^+^, K^+^-ATPase-mediated signal transduction. Na^+^, K^+^-ATPase forms a signaling complex composed of multiple structural proteins (ankyrin, caveolin), receptors (IP_3_R, EGFR), and protein and lipid kinases (Src-kinase, PI3K). Binding of ouabain to an extracellular site on Na^+^, K^+^-ATPase leads to conformational changes in the enzyme that modify its interactions with the intracellular proteins. Ouabain binding induces activation of both the PI3K/Akt pathway and the Src/EGFR/Ras/Raf/MEK/ERK kinase cascade. In turn, these events promote PLC-catalyzed production of IP_3_ and DAG, which activate IP_3_R in the ER membrane and PKC. IP_3_R, depicted as the 6-TM structure in the ER membrane, is the Ca^2+^ channel that will release Ca^2+^ (yellow hexagon) from the ER to the cytoplasm in response to an increase in IP_3_. Ankyrin is involved in organizing the Na^+^, K^+^-ATPase–IP_3_R complex. Akt, protein kinase B; DAG, diacylglycerol; EGFR, epi­dermal growth factor receptor; ER, endoplasmic reticulum; ERK, extracellular-signal regulated protein kinase; IP_3_, inositol 1,4,5-trisphosphate; IP_3_R, IP_3_ receptor; NCX, Na,Ca antiporter; PI3K, phosphatidylinositol 3-kinase; PIP2, phosphatidylinositol 4,5-bisphosphate; PIP_3_, phospha­tidylinositol (3,4,5)-trisphosphate; PKC, protein kinase C; PLC, phospholipase C; ROS, reactive oxygen species. The large red arrows depict caveolin in the flask-shaped caveolae. From reference 3, with permission.

Evidences support the notion that Na^+^, K^+^-ATPase acts as a signal transducer (40 as illustrated in Fig. 2. Na^+^, K^+^-ATPase forms a complex with Src, a non-receptor tyrosine kinase, which functions as a signal receptor for cardiotonic steroids. Src inhibition blocks many of the ouabain-activated signaling pathways. Binding of ouabain to Na^+^, K^+^-ATPase modifies the enzyme interaction with neighboring membrane proteins inducing the formation of multiple signaling modules which lead to Src kinase activation, transactivation of the epidermal growth factor receptor (EGFR) and increase formation of reactive oxygen species (41). The interaction of such signals results in the activity of several cascades, including the activation of phospholipase C (42). Multiple protein kinase cascades result activated, including mitogen-activated protein kinases and protein kinase C (PKC) isozymes in a cell-specific manner. Mitochondrial production of reactive oxygen species (ROS) is activated and intracellular calcium concentration regulated. Cross-talk among the activated pathways may lead to changes in the expression of a number of genes (43).

Signaling Na^+^, K^+^-ATPase seems concentrated in an separate pool on the plasma membrane and potential interaction between Na^+^, K^+^-ATPase and caveolins was studied, due to enzyme concentration in caveloae/rafts (41, 44).

In rat cardiac and renal cells, a cascade of events occur following ouabain interaction with a minor fraction of Na^+^, K^+^-ATPase. After sodium pump inhibition by ouabain, intracellular Na^+^ concentration increases which is followed by a gradual enhancement or oscillations in intracellular Ca^2+^ concentration. Such increase in intracellular Ca^2+^ concentration may be part of or a result of the cascade; alternatively, it may be a totally independent phenomenon (45). This process most likely involves stimulation of a clathrin-dependent endocytosis pathway leading to Na^+^, K^+^-ATPase translocation to intracellular compartments, thus suggesting a role of endocytosis in ouabain-induced signal transduction (46).

A 20-amino acid peptide (NaKtide) has been identified from the nucleotide binding domain of α1 Na^+^, K^+^-ATPase that binds and inhibits Src *in vitro*. Replacement of residues in NaKtide reduces or abolishes the inhibitory effect of the peptide on Src. However, a mutant α1 Na^+^, K^+^-ATPase that retains normal ion pumping but is defective in Src regulation has been described (47).

Although binding of endogenous cardiotonic steroids at sub-nanomolar ranges may not cause significant inhibition of Na^+^, K^+^-ATPase activity, they are able to provoke multiple protein kinase signaling events (Fig. 2). Such events include extracellular signal regulated kinase (ERK) cascades, PLC/PKC pathways, PI3K/Akt signaling and mitochondrial production of ROS (48). Na^+^, K^+^-ATPase interaction with Src forms a receptor complex for cardiotonic steroids to relay its extracel­lular binding site to intracellular signaling events. At least two pairs of domain–domain interactions are involved. The Na^+^, K^+^-ATPase second cytosolic domain binds Src SH2 domain, while the N domain directly associates with Src kinase domain and inhib­its Src activation. Cardiotonic steroids binding to Na^+^, K^+^-ATPase induces conformational changes which provide the driving force to release the Src kinase domain from the N domain. In turn, it triggers the activation of Src, which transactivates receptor tyrosines such as EGF receptor, resulting in the assembly and activation of protein kinase cascades such as Ras/Raf/ERK and PLC. As a consequence, PLC activation generates IP3 and diacylglycerol, which lead to increases in cytosolic Ca^2+^ and PKC activation. Therefore, the Na^+^, K^+^-ATPase can form a Src-coupled receptor. In this complex (Fig. 2), the Na^+^, K^+^-ATPase provides the ligand-binding site, and the associated Src acts as a signal transducer, capable of converting and amplifying the binding signal through lipid and protein kinase cascades (48).

In addition to binding Src, the Na^+^, K^+^-ATPase interacts with many other proteins. Some of them are moesin (49) and cofilin (50) which are related to actin. Other proteins are arrestin 2 and spinophilin (51) and PI3K p85 subunit (52), all involved in the modulation of Na^+^, K^+^-ATPase endo­cytosis.

The enzyme N-terminus also interacts with IP3R, Na^+^/Ca^2+^ exchanger and caveolin-1 (Fig. 2). These interactions bring the transporters and their regulatory proteins together favouring the formation of signaling complexes, allowing spatial and temporal regulation of signal transduction and coordination with trans­membrane transport. Such interactions are also impor­tant for establishing stable membrane structures such as lipid rafts (53). Moreover, there are coordinated oligomolecular complexes of Na^+^, K^+^-ATPase with gluta­mate transporters (54) and aquaporin 4 (55) among other macromolecules.

In diverse neurological pathologies it is of interest to consider the relationship between Na^+^, K^+^-ATPase with its scaffolding partners in the regulation of caveolae, cell motility and tight junctions (53, 56, 57). Other relationshps include enzyme binding with PI3K and with annexin II to regulate cell motility (56).

Activation of Na^+^, K^+^-ATPase signaling by cardiotonic steroids leads to enzyme endocytosis which most likely involves Src or PI3K (Fig. 2). Functionally, cardiotonic steroids-induced endocytosis of Na^+^, K^+^-ATPase serves as a basis to terminate the signal, to relay the signal or to target it to specific intracellular compartments (58). For detailed refer­ences and reviews of the physiological and pathological func­tions of cardiotonic steroids (7, 48).

It is known that Na^+^, K^+^-ATPase signal transduction triggers dendritic growth, and transcriptional programs dependent on cyclic AMP response element binding protein (CREB) and CRE-mediated gene expression, primarily regulated via Ca^2+^/calmodulin-dependent kinases (59).

As mentioned above, the major source of energy demand in neurons is the Na^+^, K^+^-ATPase pump that restores ionic gradients across the plasma membrane subsequent to depolarizing neuronal activity. The energy comes mainly from mitochondrial oxidative metabolism, being cytochrome oxidase a key enzyme. All cytochrome oxidase subunits are regulated by the neuron-specific factor Sp4 (59). At the same time, Sp4 regulates Atp1a1, Atp1a3, and Atp1b1 subunit genes of Na^+^, K^+^-ATPase in neurons. The expression of these genes is activity-dependent because it is up-regulated by depolarizing KCl stimulation and down-regulated by impulse blocker tetrodotoxin (59). Taken jointly, these observations indicate that Sp4 plays an important role in the transcriptional coupling of energy generation and energy consumption in neurons (59).

In the present article available experimental evidence was reviewed, which shows the alteration of Na^+^, K^+^-ATPase at CNS during aging and in pathological conditions.

## BRAIN Na^+^, K^+^-ATPase ACTIVITY DURING DEVELOPMENT AND AGING

Brain Na^+^, K^+^-ATPase increases roughly 10 times (10-fold) during development, and the increase is due to accumulation of the enzyme itself (60, 61). Thyroid hormone, known to be a regulator of brain Na^+^, K^+^-ATPase during development (62, differentially regulates enzyme isoforms (61, 63).

Na^+^, K^+^-ATPase activity in synaptosomal fractions decreases with aging in normoxic rats. The decrease in this enzyme activity by aging is more marked during adaptation to chronic intermittent severe hypoxia (64).

There are cell- and isoform- specificity alterations of Na^+^, K^+^-ATPase α isoform mRNAs in aging rat hippocampus (65) and cerebellum (66). Besides, the expression of enzyme α1 mRNA increases whereas that of α3 mRNA decreases in aging rat cerebral cortex (67).

In cerebellar Purkinje neurons of rats there is a progressive increase in resting membrane potential as well as in the depolarizing action of ouabain. Such increase correlates with that of ouabain binding sites in whole cerebellum. The increases in ouabain binding and the electrophysiological responses to ouabain seem a consequence of increases in the sodium pump. Assays with antibodies against Na^+^, K^+^-ATPase subunits show an increase in the relative amount of α3 subunit with no change in the levels of α1 or α2 subunits (68).

Studies carried out in crude microsomal preparations indicate that there is increased tendency in rat brain Na^+^, K^+^-ATPase activity from newborn to 18 days of age, suggesting that the sodium pump is mature soon after birth. No significant diferences are recorded between newborn and adult rats. Na^+^, K^+^-ATPase activity in aged rat brains is significantly lower than that at other stages of brain development. The suggestion that aged-induced decrease in brain Na^+^, K^+^-ATPase may be related to the depression of neuronal excitability and the impairement of cognitive functions has been advanced (69).

Evidence indicates that both water content and Na^+^, K^+^-ATPase activity in rat brain are significantly reduced during aging (70). Synaptosomal resting membrane potential and Na^+^, K^+^-ATPase activity decrease significantly in senescence. The decrease in phosphatidylcholine content during aging may be, at least in part, responsible for diminished enzyme activity due to alteration of lipid microenvironment. The latter, which regulates the enzyme activity, starts to change early during aging, which is followed by a decrease in Na^+^, K^+^-ATPase content. Taken jointly, these findings suggest that both changes cooperatively decrease Na^+^, K^+^-ATPase activity in senescence (71-73).

Other authors described that Na^+^, K^+^-ATPase activity (74) and ouabain binding sites (75) in human CNS as well as Na^+^, K^+^-ATPase activity, 3H-ouabain binding sites, or their affinity for ouabain in rat CNS (76) do not change with age. Curiously enough, synaptosome Na^+^, K^+^-ATPase activity in female rat brain decreases with aging whereas it remains elevated in male rat brain (77).

When brain crude synaptosomes are exposed *in vitro* to an oxidative stress by a combination of Fe^2+^ and ascorbate for up to two hours there is lipid peroxidation, extensive protein carbonyl formation and a marked decrease of Na^+^, K^+^-ATPase activity. All these changes are prevented by the presence of butylated hydroxytoluene, a chain-breaking anti-oxidant. In brain synaptosomal membrane preparations lower enzyme activity with elevated levels of lipid peroxidation products and protein carbonyls are detected in the aged rats in comparison with the young ones. These findings lead to the conclusion that age-related decline of rat brain Na^+^, K^+^-ATPase activity is most likely the consequence of enhanced oxidative damage in aging brain (78).

In the superior frontal cortex occurs a decrease in Na^+^, K^+^-ATPase α3-mRNA content per individual neuron during normal aging. This change is observed prior to the formation of Alzheimer diffuse plaques (79).

Aging induces specific changes in individual ATPases according to their subsynaptic localization. ATPase catalytic activities tend to decrease by aging. The cerebral concentration and content of somatic plasma membrane proteins increases by aging. This observation suggests that many defective noncatalytic proteins may be formed during aging, as disclosed by immunoblotting techniques (80). Na^+^, K^+^-ATPase activity in hippocampus is lower in 39 days-old rats *versus* 16 days-old rats (81).

Cognitive deficits occur in the aged brain (82). L-deprenyl protects against such deficit by improving long-term learning and memory in the aged brain. Evidences indicate that chronic deprenyl administration enhances basal electrical firing rate and the activities of Na^+^, K^+^-ATPase and PKC in CA1 and CA3 hippocampal areas, sites at which initial learning and memory processes occur (83).

Na^+^, K^+^-ATPase of synaptic plasma membranes in adult and aged animals is stimulated by ischemia. This hyperactivity is more marked in adult than in aged animals. The abnomarlities persist after 72 and 96 hours during the recirculation times, which indicate the delayed postischemic suffering of the brain. The changes in ATPase catalytic activity in synaptic membranes, modified by ischemia in presynaptic terminals, may exert an important functional role during the recovery time in cerebral tissue *in vivo*, mainly in response to noxious stimuli, particularly during the recirculation period from acute or chronic brain injury (84).

The activity of synaptosomal membrane Na^+^, K^+^-ATPase is modified by catecholamines, an effect dependent of the presence of brain soluble factors (25, 26). The effect of catecholamines and soluble factors varies with rat aging. In older rats (one year old) strong inhibitory effect is observed whereas in young rats (two weeks old) no changes in enzyme activity are recorded (28).

## Na^+^, K^+^-ATPase ACTIVITY AND BLOOD PRESSURE REGULATION

Results obtained with different experimental models suggest a relationship between blood pressure and brain Na^+^, K^+^-ATPase activity regulation. This notion is based on the effect of Na^+^, K^+^-ATPase inhibitors, termed ouabain-like substances. There are evidences which favour the notion that brain ouabain-like activity may be involved in the pressor responses to high sodium in Dahl salt-sensitive rats (85).

After aortic constriction which increases blood pressure, brain Na^+^, K^+^-ATPase expression and activity change. In whole brain, at one week after constriction all α enzyme isoforms are depressed. At four weeks, the mRNA levels of all Na^+^, K^+^-ATPase α isoforms increase in whole brain in parallel with the enhancement of α2 and α3 transcripts in hypothalamus. The initial decrease in this enzyme expression and activity may contribute to hypertension while the increase in the α2 / α3 brain expression and activity at four weeks is most likely a compensatory response to established hypertension (86).

On the other hand, examination of Na^+^, K^+^-ATPase preparations from the Milan hypertensive strain and the spontaneous hypertensive rats indicate that sensitivity to Na^+^ and inhibition curves for ouabain and mercury fail to differ from those recorded in enzyme preparations obtained from normotensive rats. These findings rule out that drastic structural alterations of the transport system occur in brain of hypertensive animals (87).

## BRAIN Na^+^, K^+^-ATPase ACTIVITY IN PATHOLOGICAL CONDITIONS

The activity of Na^+^, K^+^-ATPase is reduced or is insufficient for the maintenance of an adequate ionic balance during and after episodes of epilepsy, hypoglycemia or ischemia, as well as after the administration of excitotoxins like glutamate agonists. Na^+^, K^+^-ATPase inhibition at presynaptic level impairs the sodium gradient which drives the uptake of a variety of neurotransmitters. As a consequence, it results in the blockade of reuptake and stimulation release of glutamate and other neurotransmitters which modulate glutamate neurotoxicity (88).

Two types of brain edema may be discriminated, which are characterized by intra- or extracellular fluid accumulation. Intracellular edema occurs after cerebral ischemia, trauma, metabolic disorders and intoxications. Mechanisms involved include: a) a failure of active Na^+^ export via Na^+^, K^+^-ATPase due to energy shortage, b) an enhanced Na^+^ permeability, or c) the activation of Na^+^ driven membrane pumps. Extracellular edema occurs in brain tumors, infections, trauma and hypertensive crisis. It is caused by damage of the blood-brain barrier and is accompanied by protein-rich fluid (89).

Administration of Na^+^, K^+^-ATPase inhibitors ouabain, digoxin and digitoxin antagonize the antinociceptive effect of morphine in mice. This effect is not attributable to an interaction at opioid receptors. The suggestion that the activation of Na^+^, K^+^-ATPase plays a role in the supraspinal, but not spinal, antinociceptive effect of morphine has been formulated (90).

## ISCHEMIA

Acute cerebral ischemia induced by middle cerebral artery occlusion leads to changes in Na^+^, K^+^-ATPase activity, water content as well as Na^+^ concentration. The cellular edema seems associated with impaired membrane pump function (91). Focal cerebral ischemia is associated with a decrease in Na^+^, K^+^-ATPase activity and in alteration in affinity of enzyme sites for ouabain. Only two sites for ouabain are detected though all three α isoforms are present. No changes in protein and mRNA expression of α or β isoforms are recorded. The suggestion that ischemia leads to intrinsic modifications in Na^+^, K^+^-ATPase, which result in alteration of membrane integrity and / or association of the α isoforms has been advanced (92, 93).

Aplication of single transient forebrain ischemia in adult rats causes inhibition of Na^+^, K^+^-ATPase activity in hippocampal and cerebral cortex membrane fractions. Ischemic preconditioning prevents the inhibitory effect of ischemia / reperfusion on Na^+^, K^+^-ATPase activity. It has been hypothesized that the maintenance of Na^+^, K^+^-ATPase activity afforded by preconditioning is related to neuronal protection (94).

In an experimental model of ischemic preconditioning carried out in hippocampal slice cultures two regulators has been identified: protein kinase M zeta and the Na^+^, K^+^-ATPase. Whereas at short time (two hous) following preconditioning there is neither alteration of the regulators or neuroprotection, at longer period (24 hours) there is enhancement of protein kinase M zeta and the Na^+^, K^+^-ATPase. Most interestingly, inhibition of α1 and α2 / α3 Na^+^, K^+^-ATPase isoforms blocks neuroprotection following ischemic preconditioning. The roles of the mentioned regulators in persistent neuroprotective mechamism of ischemic preconditioning have been advanced. These findings lead to the conclusion that increased Na^+^, K^+^-ATPase activity protects slice culture neurons from hypoxia-hypoglycemia (95).

It is known that ouabain inhibits Na^+^, K^+^-ATPase activity within the 10^-7^-10^-3^ M concentration range but stimulates this enzyme activity at lower ranges (96). The stimulatory effect of cardiotonic steroids is recorded in hippocampal slice cultures *in vitro* as well as in hippocampus *in vivo*. Ouabain protects slice culture neurons from experimental ischemia at concentrations that enhanced Na^+^, K^+^-ATPase. These observations suggested that the protective effect of ouabain is due to increased Na^+^, K^+^-ATPase activity (97).

A hypoxic precondition induces neuroprotection against transient global ischemia in adult rats. This result is due to enhanced recovery of Na^+^, K^+^-ATPase activity by preservation of protein levels of enzyme α1 subunit and reduced DNA fragmentation after ischemia (98).

## BRAIN INJURY

Cryogenic lesion to brain produces different changes in water content and Na^+^, K^+^-ATPase activity according to rat age. The increase in water content was larger in adult rats whereas impairement of Na^+^, K^+^-ATPase activity is more pronounced in aged animals (70).

In experimental traumatic brain injury there is a decrease in brain Na^+^, K^+^-ATPase activity concomitant with an increase in the levels of lipid / protein oxidation. At the same time, an alteration in membrane fluidity and neuronal excitability is observed (99).

Pearson´s correlation analysis discloses strong correlation of myeloperoxidase increase with Na^+^, K^+^-ATPase inhibition in sedentary rats. Previous running exercise (4 weeks) protects against fluid percussion brain injury (FPI)-induced and motor function impairement and fluorescein extravasation. Physical training is effective against myeloperoxidase activity increase and Na^+^, K^+^-ATPase activity decrease after FPI. This protection correlates with myeloperoxidase activity decrease and indicates that the alteration of cerebral inflamatory status profile observed by previous physical training diminishes initial damage and limits long-term secondary degeneration after traumatic brain injury (100).

## DEPRESSION AND MOOD DISORDERS

Diverse evidences suggest the involvement of Na^+^, K^+^-ATPase dysfunction in the pathophysiology of unipolar and bipolar disorder (101). A model for mood cycle regulation has been proposed involving steroid hormones by means of Na^+^, K^+^-ATPase regulation; this hypothesis contends that steroid hormones decreases Na^+^, K^+^-ATPase activity in hypothalamus, directly or through their conversion into digitalis-like compounds, which in turn, stimulate beta-endorphin secretion, normally leading to elevated mood (102).

Evidences indicate that depressive disorders result from a combination of inherited susceptibility genes and environmental stress (103). A reduction in Na^+^, K^+^-ATPase expression and function is associated with depressive disorders in humans (104-107) as well as in animal models of depression (108, 109). In a model of depression employing female rat reduction in hippocampal synaptic membrane Na^+^, K^+^-ATPase activity has been observed. Treatment with fluoxetine enhances the enzyme activity and reverses the effect of stress, suggesting that altered Na^+^, K^+^-ATPase activity may well be involved in the pathophysiology of depression in patients (108).

There is a reduction of Sp4 protein levels in the cerebellum and prefrontal cortex of bipolar disorder subjects, supporting a possible role of this factor in the pathogenesis of the disease. Besides, Sp4 stability is regulated by neuronal activity and lithium stabilizes Sp4 protein. These observations suggested that normalization of Sp4 levels could contribute to treatment of affective disorders (109).

Na^+^, K^+^-ATPase α3 heterozygous mice develop enhanced depression-like endophenotypes in a chronic variable stress paradigm in comparison to wild-type littermates (Atp1a3(+/+)). In Atp1a3(+/+) mice chronic variable stress fails to decrease Na^+^, K^+^-ATPase activity. Therefore, a mutation that diminishes neuronal Na^+^, K^+^-ATPase activity exacerbates depression induced by stress. An interesting correlation between Na^+^, K^+^-ATPase activity and mood in unipolar and bipolar disorders has been advanced (110).

On the other hand, inhibition of calcineurin, which leads to Na^+^, K^+^-ATPase activity decrease, induces depressive-like behavior via mTOR signaling pathway (111).

## MANIA

Findings suggest a relationship between Na^+^, K^+^-ATPase α3 subunit and mania. Intracerebroventricular administration of ouabain induces behavioral changes in a rat model of mania (112-115).

Regarding to this point, it is of interest to consider the involvement of agrin, a proteoglycan which behaves as an antagonist for Na^+^, K^+^-ATPase α3 subunit and exerts regulatory properties of brain function. Through its inhibition of Na^+^, K^+^-ATPase α3 subunit, agrin is important in Ca^2+^ homeostasis and neuronal activity (116).

Myshkin mice which carry an inactivating mutation in the Na^+^, K^+^-ATPase α3 subunit display a behavioral profile very similar to bipolar patients in the maniac state. These mice present increased Ca^2+^ signaling in cultured cortical neurons and phosphoactivation of ERK and Akt in hippocampus. Most interesting, specific ERK inhibitor SL327 and transgenic expression of a functional Na^+^, K^+^-ATPase α3 subunit rescue the mania-like phenotype of Myshkin mice (117). The expression of agrin has been related to Myshkin mice behaviour and agrin has been proposed as a potential therapeutic target for the treatment of mania and other neurological pathologies related to reduced Na^+^, K^+^-ATPase activity and neuronal hyperexcitability (118).

## STRESS

;Na^+^, K^+^-ATPase activity in rat CNS is influenced by neonatal handling. Enzyme activity decreases in hippocampus but increases in the amigdala of neonatally-handled rats. Chronic variable stress diminishes Na^+^, K^+^-ATPase activity in hippocamppus, amigdala and parietal cortex. Therefore, early handling increases the ability to cope with chronic variable stress in adulthood. At that stage, animals show less susceptibility to neurochemical features related to depression, supporting the relevance of the precocious environment to vulnerability to psychiatric disorders in adulthood (119).

Exposure of rats to repeated restraint stress reduces the levels of Na^+^, K^+^-ATPase in brain structures and changes both short- and long-term memory, learning and exploratory response (120).

## NEURONAL HYPEREXCITABILITY AND EPILEPSY

The common pathogenetic factor for a congenital status convulsivus has been attributed to a defect of astrocyte Na^+^, K^+^-ATPase (121).

At the time of convulsions induced by pentylenetetrazol a considerable swelling of astroglial cells occurs as disclosed at the electron microscope level. Concomitantly, Na^+^, K^+^-ATPase activity in rat cerebral cortex homogenates is higher than that in the controls. At variance, the activities of Mg^2+^-ATPase, K^+^- and Mg^2+^-*p*-nitrophenylphosphatases remain unaltered by the treatment (122).

There is an increase of Na^+^, K^+^-ATPase activity in the primary focus of cat cerebral cortex produced by acute freeze lesions. Both K^+^ and phenytoin dephosphorylating influences are decreased in primary and secondary foci of acutely lesioned cats. In chronic cats, the dephosphorylating step of Na^+^, K^+^-ATPase catalytic subunit recovers a normal affinity to K^+^ whereas its sensitivity to phenytoin remains diminished. It has been advanced that such differences in K^+^ and phenytoin influences on brain Na^+^, K^+^-ATPase between control *versus* epileptic cortex might be responsible for the ictal transformation and seizure spread (123).

Potential regulatory mechanisms for extracellular K^+^ concentration during spontaneous recurrent epileptiform activity was studied. During hyperactivity induced in the dentate gyrus of hippocampal slices from rats by perfusion with potassium, the effect of several drugs was tested. Results obtained in this experimental model with tetrodotoxin, furosemide, barium and cesium salts, and ouabain, lead to the conclusion that potassium redistribution by glia only plays a minor role whereas the major regulator of extracellular K^+^ concentration seems to be ion uptake via Na^+^, K^+^-ATPase, most likely the neuronal one (124).

Partial inhibition of Na^+^, K^+^-ATPase with the low affinity cardiac glycoside dihydroouabain produces neuronal hyperexcitability in CA1 hippocampal slices. Such effect is attributable to reduced GABAergic potentials and enhanced coupling between excitatory postsynaptic potentials and spike firing (125).

A1A2 Na^+^, K^+^-ATPase mutations occur in familial hemiplegic migraine type 2 (FHM2) (126). Three putative A1A2 mutations have been identified. Among them, those termed D718N and P979L, may predispose to seizures and mental retardation (127).

The activity of Na^+^, K^+^-ATPase in hippocampus increases after multiple status epilepticus induced by pilocarpine in developing rats. The increases are notably higher when the assays are carried out at 30 days *versus* 7 days following the injections (81).

Familial hemiplegic migraine is a form of migraine with aura associated with several neurological signs, including epileptic seizures (128). The sporadic form of hemiplegic migraine presents with the same symptoms (129). In a case of a young hemiplegic migraine patient epileptic seizures occurred in the childhood which was controlled with drug therapy. This sporadic case carries a nonsense muration p.Tyr1009X in the ATP1A2 gene, leading to a truncated α2 subunit of the Na^+^, K^+^-ATPase thus lacking the last 11 amino acids. This mutation confirms the role of this gene in forms of hemiplegic migraine associated with epilepsy (130).

It is known that the two autosomal dominantly inherited neurological diseases: familial hemiplegic migraine type 2 and familial rapid-onset of dystonia-parkinsonism are caused by mutations of specific α subunits of Na^+^, K^+^-ATPase. Patients with classical symptoms of these diseases suffer other manifestations, such as epileptiform activity. Mouse models targeting a specific α isoform produce comparable phenotypes consistent with classical symptoms observed in familial hemiplegic migraine type 2 and familial rapid-onset of dystonia-parkinsonism patients (131).

Dopamine oxidation products exert diverse damaging effects on brain subcellular components. It is known that dopamine causes *in vitro* neuronal Na^+^, K^+^-ATPase inhibition (25, 132). The inactivation of neuronal Na^+^, K^+^-ATPase by dopamine may lead to various toxic sequelae with potential implications for dopaminergic cell death in Parkinson´s disease (132).

It is known that ouabain-induces hyperactivity in rats (112-115) and ouabain binding to Na^+^, K^+^-ATPase affects *in vitro* signaling molecules, most likely ERK1/2 and Akt, which promote protein translation (43, 133).

Ouabain injection to rats leads to hyperactivity and increased phosphorylation levels of mTOR, p70S6K, S6, elF4B and 4E-BP. Findings suggest that ouabain administration induces activation of the protein translation initiation pathway regulated by ERK1/2 and Akt, and prolonged hyperactivity in rats (134).

It is known that traumatic brain injury is a major cause of acquired epilepsy. Exercise training is effective against several neurochemical alterations including the inhibition of Na^+^, K^+^-ATPase activity after fluid percussion injury (135).

## ALZHEIMER DISEASE

Alzheimer disease is a neurodegenerative disorder characterized clinically by progressive memory and cognitive dysfunction associated to neuronal loss. Autopsy from patients clinically and histopathologically diagnosed as having Alzheimer disease exhibit several criteria which include the accumulation of amyloid-beta (Aβ) peptide plaques and neurofibrillary tangles (NFTs) in the brain. The levels of Aβ and tau/phospho-tau in the cerebrospinal fluid are associated with this pathology (136, 137).

There is a marked decrease in brain ouabain binding in patients with Alzheimer disease in comparison with age matched controls, particularly in the cerebral cortex (75). In relation with impaired neuronal function, the activity of Na^+^, K^+^-ATPase is significantly lower in the brains of patients with Alzheimer´s disease than in the brains of normal controls (138).

The exposure of cultured rat hippocampal neurons to Aβ peptide leads to selective reduction of Na^+^, K^+^-ATPase activity which precedes the loss of calcium homeostasis and cell degeneration. The treatment fails to impair the activity of Mg^2+^-dependent ATPase or that of the Na^+^/Ca^2+^ exchanger. Inhibition of Na^+^, K^+^-ATPase activity with ouabain is sufficient to induce elevation of Ca^2+^ and neuronal injury. Neuronal degeneration produced by ouabain seems to be due to an apoptotic form as indicated by nuclear condensation and DNA fragmentation. Besides, the exposure of synaptosomes isolated from postmortem human hippocampus to Aβ specifically reduces Na^+^, K^+^-ATPase and Ca^2+^-ATPase activities, without altering the activity of Mg^2+^-dependent ATPase or that of the Na^+^/Ca^2+^ exchanger. These findings lead to the suggestion that impairement of ion-motive ATPase activities may be important for the pathogenesis of neuronal injury in Alzheimer disease (139).

Amyloid impairs glucose transport in hippocampal and cortical neurons, an effect which involves membrane lipid peroxidation (140). This peroxidation may well explain the reduction of Na^+^, K^+^-ATPase activity. Oxidative stress, mitochondrial dysfunction, and impairment of Na^+^, K^+^-ATPase activity in hippocampal neurons induced by amyloid are attenuated by basic fibroblast growth factor (bFGF). Na^+^, K^+^-ATPase activity is significantly reduced following exposure to Aβ (25-35) toxicity in control hippocampal cultures but not in cultures pre-treated with bFGF (141). Impairement of Na^+^, K^+^-ATPase activity by amyloid beta-peptides in rat hippocampal cultures (141) is not readily reversible and occurs only after amyloid incubation with intact hippocampal slices but not with disrupted membranes (142).

In superior frontal cortex from Alzheimer disease subjects there occurs an increase in α1-mRNA Na^+^, K^+^-ATPase, most likely related to enhanced reactive gliosis. At the same time, there is a decrease in Na^+^, K^+^-ATPase α3-mRNA. The suggestion that the declines in α3-mRNA *per neuron* which occurs in normal aging may predispone to or potentiate Alzheimer disease pathogenesis has been advanved (79). Na^+^, K^+^-ATPase activity and enzyme α3 subunit are lower in Alzheimer´s disease brains *versus* control brains. In contrast, the amount of protein disulfide isomerase, which is one of the house keeping membrane proteins, fails to differ between groups (143).

Evidences support a key role for protein phosphorylation in both normal and pathological actions of Aβ and the formation of NFTs. Protein kinases are involved in the actions of tau or Aβ. However, protein phosphatases such as serine/threonine protein phosphatases that reverse the actions of protein kinases are important likewise in the pathology of Alzheimer´s disease (144).

Calcineurin may play an especial role in Alzheimer´s disease because it modulates Na^+^, K^+^-ATPase activity. Calcineurin protein levels are inversely correlated with dementia severity and Braak tangle stage in the brain of patients with Alzheimer disease (145). Soluble Aβ oligomers activate calcium-dependent phosphatase calcineurin (PP2B) which in turn activates the transcriptional nuclear factor of activated T cells (NFAT). Aβ deposits are ameliorated by calcineurin inhibition, supporting the notion the calcineurin-NFAT are aberrantly activated by Aβ and that calcineurin-NFAT activation is responsible for disruption of neuronal structure close to the plaques. The conclusion that neurodegeneration in Alzheimer disease, at least partially, occurs by activation of calcineurin followed by NFAT-mediated downstream cascades has been advanced (146).

**Figure 3:**
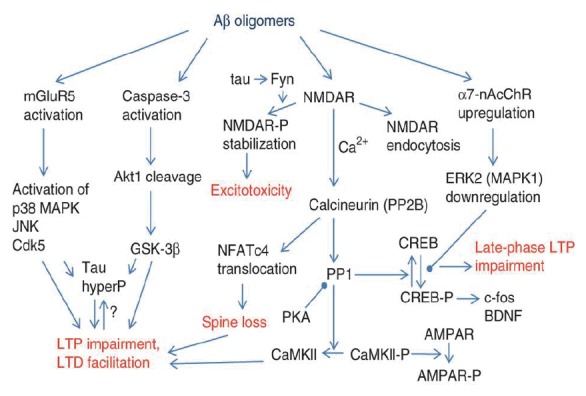
Molecular mechanisms proposed for Aβ synaptotoxicity. The diagram summarizes some pathways which have been invoked in several experimental paradigms for synaptotoxicity. Aβ directly or indirectly modifies glutamate receptor-dependent cascades, which in turn lead to LTP impairement and LTD facilitation. Activation of calcineurin and NFATc4 induces dystrophic changes in neurites, and calcineurin-dependent dephoshorylation of CaMKII impairs the induction of AMPAR-based LTP in hippocampus. Other changes induced by Aβ include upregulation of α7-nAcCh receptor, tau phosphorylation and caspase-3 activation which also lead to LTP impairement. mGluR5, metabotropic glutamate receptor 5; ERK2, extracellular signal-regulated kinase 2; MAPK, mitogen-activated protein kinase; JNK, c-Jun N-terminal kinase; Cdk5, cyclin-dependent kinase 5; GSK-3β, glycogen synthase kinase-3β; Akt1, serine-threonine protein kinase 1; NMDAR, *N*-methyl-D-aspartate receptor; AMPAR, α-amino-3-hydroxyl-5-methyl-4-isoxazolepropionate receptor; CaMKII, calmodulin kinase II; PKA, protein kinase A; PP1, protein phosphatase 1; NFATc4, nuclear factor of activated T-cells; α7-nAcChR, α7-nicotinic acetylcholine receptor; BDNF, brain-derived neurotrophic factor: CREB, cyclic AMP response element binding protein. For a complete description, see reference 147. From reference 147, with permission.

Diverse evidences support the notion that Aβ oligomers are the drivers of neurodegeneration which occurs in Alzheimer disease (147). Some of the pathways involve the activation of glutamatergic metabotropic mGluR5 receptor (148) and that of caspase-3 which leads to Akt1 cleavage (149). Other interactions with Na^+^, K^+^-ATPase activity include ERK2 downregulation as well as the N-methyl-D-aspartate (NMDA) ionotropic glutamate receptor activation (150, 151), closely related to Na^+^, K^+^-ATPase activity (Fig. 3).

In fact, results recorded in several experimental models suggest a close relationship between the activity of Na^+^, K^+^-ATPase and NMDA receptor in intact cells (152-154). Aβ oligomers modify the activity of calcineurin (146), known to enhance Na^+^, K^+^-ATPase activity by its dephosphorylation (15, 16). Besides, Ca^2+^ influx through the NMDA receptor activates calcineurin and protein phosphatase 1, thus modifying Na^+^, K^+^-ATPase activity (155).

## Na^+^, K^+^-ATPase INVOLVEMENT IN LEARNING AND MEMORY

Inhibition of Na^+^, K^+^-ATPase activity produces edema and cell death at CNS level and also impairs learning and memory. Several sex steroid hormones protect against neuronal cell damage and disfunction of learning and memory. Accordingly, 17beta-estradiol and testosterone ameliorate amnesia induced by ouabain, a non-genomic effect which is independent of radical scavenging action (156).

It is known that cognitive deficits occur in the aged brain. L-deprenyl protects against such deficit by improving long-term learning and memory in the aged brain. Evidences indicate that chronic deprenyl administration enhances basal electrical firing rate and the activities of Na^+^, K^+^-ATPase and PKC in CA1 and CA3 hippocampal areas, sites at which initial learning and memory processes occur (83).

## DIABETES

It is known that insulin is one of the many hormones which regulate the activity of Na^+^, K^+^-ATPase. Alteration of this enzyme activity has been associated to diverse diabetic complications (157). Insulin enhances the activity of membrane-bound ATPase isolated from rat brain (158), an effect which is dependent on both: the experimental assay condition and the integrity of some cell membranes (159). Relatively high hormone concentrations are required to obtain maximal insulin effect. Such effect most likely involves the high affinity enzyme isoform (160).

Streptozotocin administration to laboratory animals exerts a specific toxic effect to the pancreatic beta cells, which undergo their destruction by necrosis inducing a diabetic state (161). This state is associated with a reduction in brain Na^+^, K^+^-ATPase activity (162-164). Changes recorded in α3 isoform expression and Na^+^, K^+^-ATPase activity are dependent on the time elapsed after drug administration (162). Findings suggested that the drug may alter first *per se* isoform expression. In time, the lower enzyme activity may be a consequence of the hyperglycemic diabetic state (165).

## CANCER

Evidences showing the involvement of Na^+^, K^+^-ATPase in regulating carcinoma cell motility have been reported (56). Na^+^/K^+^ pump is directly involved in the migration of cancer cells in general and of glioma cells in particular. The Na^+^, K^+^-ATPase α1 subunit is highly expressed in glioma cells *versus* normal brain tissue and has been propossed as a new target for malignant glioma treatment (166). Glioblastoma patients resistant to chemotherapy and whose tumors over-express Na^+^, K^+^-ATPase α1 subunit could benefit from a treatment using ligands with higher binding affnity for the enzyme α subunit (167).

It has been reported that the frecuency of strong FXYD3 expression is higher in the primary tumors in comparison to normal brain tissue. Increases in FXYD3 expression are higher in females than in males and in multiple site gliomas than in single sites. This result suggests that FXYD3 expression may be involved in glioma development, spetially in multiple gliomas and female patients (168).

## CONCLUDING REMARKS AND FUTURE DIRECTIONS

Diverse lines of evidence lead to the notion that Na^+^, K^+^-ATPase (sodium pump) exerts vital roles in normal brain function. Na^+^, K^+^-ATPase is concentrated in the synaptic membranes where it participates in diverse important reactions involved in neurotransmission. For this reason, a fine tuning of this enzyme activity is essential. Inhibition of Na^+^, K^+^-ATPase by ouabain impairs several biochemical and physicochemical activities. To illustrate, incubation of isolated synaptosomes with ouabain inhibits oxidative metabolism, the synthesis of high energy compounds, proteins and lipids, as well as the uptake of neurotransmitters and their precursors. Likewise, ouabain blocks both potassium uptake and sodium release, which are required to restore ionic equilibria after the passage of nervous impulse (169). These facts indicate that it is important that Na^+^, K^+^-ATPase could function adequately. Otherwise, its malfuctioning could obviously lead to diverse alterations of neuronal behaviour.

It should be considered that Na^+^, K^+^-ATPase changes recorded in a given pathological condition may involve a direct effect on enzyme expression or activity. Alternatively, they may be due to indirect effects following the alteration of neurotransmitter receptors which are closely located in the synaptic membranes.

There are domain-specific interactions which make the enzyme Na^+^, K^+^-ATPase an important scaffold in forming signaling microdomains. By direct or indirect interactions, it is able to modify numerous enzymes and intracellular factors which are involved in signaling pathways, including Src and many other proteins. Some of them modulate Na^+^, K^+^-ATPase endocytosis, are related to actin, or include IP3R, Na^+^ / Ca^2+^ exchanger or caveolin-1. Other interactions favour the formation of stable membrane structures such as lipid rafts and oligomolecular complexes with gluta­mate receptors and transporters and aquaporin 4, among other macromolecules.

Regarding neuronal excitability with Na^+^, K^+^-ATPase activity, there is the notion that its malfunctioning is currently associated with neuronal hyperexcitability (81). An ultraslow, minute-long afterhyperpolarization in network neurons following locomotor episodes has been described. It is mediated by an activity- and sodium spike-dependent enhancement of electrogenic Na^+^/K^+^ pump function. The ultraslow hyperpolarization seems related to short-term memory of neural network function *via* activity-dependent potentiation of Na^+^/K^+^ pump function (170).

On the other hand, the activation of the limbic-hypothalamic-pituitary-adrenal axis and the release of glucocorticoids are fundamental for the adaptive response and immediate survival of an organism in response to acute stimuli. However, high levels of glucocorticoids in brain may lead to neuronal injury and decreased Na^+^, K^+^-ATPase activity, affecting neurotransmitter signaling, neural activity and animal behavior (120).

Studies with existing cardiac glycosides or other ouabain-like substances termed endobains (27) could provide an adecuate path toward clinical tests for ischemic stroke. Such studies can play a role in the identification of new candidate drugs as well as drug targets for the treatment of diseases for which adequate therapeutic pathways are not available nowadays.

The availability of a wide spectrum of Na^+^, K^+^-ATPase enzyme isoforms mutations represents a valuable tool to disclose the mechanisms involving this enzyme in diverse neurological pathologies. Moreover, the use of mouse models offers broad potentials for future research concening migraine and dystonia-related diseases (131).

It is known that ifenprodil (a NMDA receptor antagonist) restores both Na^+^, K^+^-ATPase expression and GDNF-evoked Ca^2+^ signalling Na^+^, K^+^-ATPase expression in inflammation-pretreated astrocytes (171). This observation offers another potential relationship to take into account in neurological diseases involving Na^+^, K^+^-ATPase expression and NMDA receptor deficiency.

As mentioned above, evidences show that in Alzheimer disease patients Na^+^, K^+^-ATPase activity is lower than in control patients. On the other hand, Sp4 levels are dramatically increased and associated with neurofibrillary tangles and pathological tau presence in neurons of the CA1 hippocampus region and entorhinal cortex of Alzheimer disease patients (171). Taken into account that Sp4 regulates the expression of Na^+^, K^+^-ATPase subunit genes in neurons (59), the sodium pump may be a target in translational medicine.

Regarding potential brain function recovery after trauma, it is of interest to recall that physical exercise produces favorable effects in neuroreabilitation after traumatic brain injury (100).

It is tempting to advance that the employ of pharmaceuticals to block Na^+^, K^+^-ATPase inhibition by endogenous substances or to enhance Na^+^, K^+^-ATPase activity may be of help to treat depressive disorders or to avoid depressive disorders in susceptible individuals.

## ABBREVIATIONS

Aβ peptide: amyloid-beta peptide
Akt, protein kinase B (PKB): serine/threonine-specific protein kinase
Atp1a1, Atp1a3, and Atp1b1: Na^+^, K^+^-ATPase mutants
bFGF: basic fibroblast growth factor
CNS: central nervous system
CREB: cyclic AMP response element binding protein
DAG: diacylglycerol
DARPP-32: cAMP-regulated phosphoprotein of 32 kDa
4E-BP: translational regulator protein
elF4B: initiation factor
EGF: epi¬dermal growth factor
EGFR: epi¬dermal growth factor receptor
ER: endoplasmic reticulum
ERK: extracellular-signal regulated protein kinase
FHM2: familial hemiplegic migraine type 2
FPI: fluid percussion brain injury
FXYD: Na^+^, K^+^-ATPase γ subunit
IC50: half maximal inhibitory concentration
IP_3_: inositol 1,4,5-trisphosphate
IP3R: IP3 receptor
LTD: long term depression
LTP: long term potentiation
Mg^2+^-ATPase: magnessium-activated adenosine 5’-triphosphatase
MEK: mitogen-activated protein kinase kinase (MARK kinase)
NaKtide: Na^+^, K^+^-ATPase 20-amino acid peptide
Na^+^, K^+^-ATPase: sodium- and potassium-activated adenosine 5’-triphosphatase
NCX: Na^+^, Ca^2+^ antiporter
NFAT: nuclear factor of activated T cells
NFTs: neurofibrillary tangles
NMDA receptor: N-methyl-D-aspartate ionotropic glutamate receptor
PP2B: calcium-dependent phosphatase calcineurin
PI3K: phosphatidylinositol 3-kinase
PIP_2_: phosphatidylinositol 4,5-bisphosphate
PIP_3_: phospha¬tidylinositol (3,4,5)-trisphosphate
PKB: protein kinase B
PKC: protein kinase C
PLC: phospholipase C
Ras proteins: small GTPases that regulate cell growth, proliferation and differentiation
Raf kinases: family of three serine/threonine-specific protein kinases
ROS: reactive oxygen species
SL327: specific ERK inhibitor
Sp4: sprouty 4 factor
Src-kinase: non-receptor tyrosine kinase
mTOR: mammalian target of Rapamycin, serine/threonine kinase
p70S6K: serine/threonine protein kinase that phosphorylates the ribosomal S6 subunit
S6: a component of the 40S ribosomal subunit.
